# Lipids from Oilcakes—High Quality Ingredients for Functional Food Products

**DOI:** 10.3390/molecules30173640

**Published:** 2025-09-06

**Authors:** Ancuța Petraru, Sonia Amariei, Lacrimioara Senila

**Affiliations:** 1 Faculty of Food Engineering, Stefan cel Mare University, 720229 Suceava, Romania; sonia@usm.ro; 2 National Institute for Research and Development of Optoelectronics INOE 2000, Research Institute for Analytical Instrumentation, 67 Donath Street, 400293 Cluj-Napoca, Romania; lacri.senila@icia.ro

**Keywords:** fatty acids, oilseeds, oilcakes, nutritional indexes

## Abstract

Fatty acids (FAs) are vital for human nutrition and are classified into three categories (saturated, unsaturated, and trans). FAs have different physiological effects and can contribute to health problems in different ways. By-products from the oil industry are rich in bioactive compounds. These make them useful for further utilization in food formulation. There is a quantity of residual oil in the oilcake. Analysis of the fatty acid composition shows that unsaturated fatty acids are predominant. The predominant fatty acids in oilcakes are arachidic (sunflower), oleic, elaidic (flax), linoleic (LA), and linolelaidic (hemp, rape, and sesame) acids. The favorable and ideal (within the regulatory recommendations) results for the n-6/n-3 ratios of 3:1 indicate the high nutritional profile with beneficial effects for the human body of the oilcakes. The hypocholesterolemic/hypercholesterolemic for all samples ranged from 4.52 to 116.06, while atherogenicity and thrombogenicity indexes ranged from 0.01 to 0.3. This is in line with the favorable values found in the literature benchmarks.

## 1. Introduction

Nowadays, our society is facing two main challenges, resource exhaustion and waste accumulation, leading to higher raw material costs and costly, restrictive waste disposal legislation. The European Union Directive regarding waste management includes a five-step approach: prevention, reuse, recycling, recovery, and last disposal [1]. Following these recommendations, food waste from different agri-food industries becomes interesting as cheaper sources of potential functional compounds that make them useful in the pharmaceutical and nutraceutical fields [2,3]. The main organic waste is generated from three industrial sectors: oil, agriculture and food [4]. From post-harvest to distribution, the highest losses are recorded in root and oilseed crops (25%), followed by fruits and vegetables (22%), meat and animal products (12%), and cereals and legumes (9%) [5].

Generally, the by-products resulting from the oil industry are recognized for their high protein and fiber content, but their residual lipidic fraction and FA profile have received limited attention. This limitation becomes of interest when using these by-products as novel food ingredients for food formulations. Creating novel food ingredients that can address sustainability goals and nutritional challenges is a key priority in food science and technology. As the demand for healthy, minimally processed, and organic foods increases, so does the interest in alternative plant-based ingredients [6]. Oilcakes possess valuable techno-functional properties (water/oil absorption, swelling, and emulsifying abilities), which make them appealing for use in food formulation because they improve the structural, rheological, and sensor characteristics [7]. Despite this, the by-products remain underutilized. Other potential applications include their use in animal feed and the development of biodegradable materials and functional ingredients [8,9,10]. Proper processing and detoxification could unlock their nutritional and functional value, supporting food security and circular economy initiatives [11].

Dietary lipids are important sources of energy, and other bioactive compounds (fatty acids, fat soluble vitamins, phenolic lipids, carotenoids, and phytosterols) play key roles in numerous biological functions [12,13]. Fatty acids (FAs) are the main components of dietary lipids and are generally derived from phospholipids and triglycerides [14]. FAs are organic acids containing at least one carboxylic group attached to a long carbon chain (from two to thirty-two carbon atoms) [15]. Depending on the carbon bonds, the fatty acids can be divided into: saturated (SFA, single bond), monounsaturated (MUFA, one double bonds), or polyunsaturated (PUFA, two or more double bonds) [16].

Over time, there has been a lot of emphasis on the continued demonization and general avoidance of dietary fats. Unfortunately, this has led to the unintended consequences of an increasing carbohydrate and sugar intake [17]. Therefore, it is important to pay more attention to the quality of fats than their quantity.

FAs play critical roles (positive or negative) in human metabolism, health, and a wide array of diseases (in prevention and treatment) [18]. In particular, PUFA have a positive effect on cardiovascular [19,20,21], neurological [22,23], allergic [24], and non-alcoholic fatty liver [25] diseases. In PUFA classes, the major acids are α-linolenic (ALA, n-3) and LA (n-6), defined as essential FAs since they are obtained largely from the diet and cannot be synthesized in the human body [26,27].

While UFAs have positive effects on human health, numerous studies have shown that saturated and trans FAs increase the risk of atherosclerotic cardiovascular and cognitive diseases such as Alzheimer’s [28,29]. Coronary heart disease occurs when the percentage of low-density cholesterol, called bad cholesterol, increases in the bloodstream and the percentage of high-density cholesterol, called good cholesterol, decreases [30]. In conclusion, the balance between different types of FA is pivotal. The main sources of UFA are fatty fish, nuts, seeds, and plant-based oils [31].

The main objective of this study was to provide a comprehensive overview of the lipid profile of the most representative seeds (sunflower, hempseeds, rapeseed, sesame, flaxseed, and walnut) and the resulting cold-pressed oilcakes. The aim is to focus on the contribution of each fatty acid to human health in the prevention and monitoring of chronic diseases. In addition, this study aimed to demonstrate the possibility of successfully integrating oilcakes into the human diet as functional foods or supplements.

## 2. Results

### 2.1. Oilseeds

Among seeds, the lowest lipid concentration was observed for flax seeds (FS), and the highest for sunflower seeds (SFS) and walnut kernels (WK).

With the exception of flax seeds, in all tested seeds, PUFA were dominant, while the amount of SFA was the lowest. The proportions of fatty acids depend on the seed species and vary significantly among the samples tested.

The percentage of SFA in oilseeds varied between 1.73% (WK) and 25.22% (SFS) of total fatty acids. Caprylic, myristic, pentadecanoic, and stearic acids were present in all the samples. Pentadecanoic acid was the dominate SFA in HS (hemp seeds), RS (rape seeds) and FSs. Arachidic, palmitic, and heptadecanoic acids were dominant in the other oilseeds (SF, SS—sesame seeds and WK). Capric, lauric, tricosanoic, and lignoceric acids were not confirmed in SS and WK, but present in the others, with a significantly higher amount in RS. The share of heneicosanoic acid was the highest in FS, whereas in SFS and HS was not detected. The lowest percent for a SFA (eicosadienoic acid, 0.14%) was detected in HS. RS had the highest content of SFA with an even number of carbons in the chain, while FS contained the highest content of SFA with an odd number of carbons in the chain.

The MUFA content ranged from 17.44% (SFS) to over 40.63% (SS) of the total FA. Myristoleic, palmitoleic, erucic, oleic, and elaidic acids were the main MUFA determined in all tested seeds, with the highest levels for the last two FAs. Among the odd-chain MUFA only cis-10-pentadecanoic and cis-10 heptadecanoic were detected. The first was found in SFS, RS, and SS, while the second was found in the other samples (HS, FS, and WK).

The share of PUFA in the oilseeds samples was from 25.35% (FS) to 67.92% (WK). LA, linoelaidic, ALA, cis-11,14-eicosadienoic, cis-8,11,14-eicosatrienoic, cis-11,14,17-eicosatrienoic, arachidonic, docsa-hexanoic, and nervonic acids were detected in all the samples. The highest concentrations of LA and linoelaidic acids were obtained for WK; its share was about 66%. However, these acids were dominant in most of the examined seeds, constituting over 40%. In the category of PUFA n-3, ALA predominates in FS (2.46%) and SFS (1.25%).

Fat contents and fatty acid profiles of the tested seeds samples varied significantly (*p* < 0.05%). The values are presented in Table 1.

### 2.2. Oilcakes

The lipid contents in oilcakes were significantly lower than in seeds, and their contents varied from 9.63% (hempseed oilcake, HSOC) and 24.66% (SOC, sesame oilcake).

The SFA content varied between 12.09% and 55.84%. The highest content was determined in flaxseed oilcake (FSOC), while the lowest was found in sunflower oilcake (SFOC). In terms of individual SFA, all samples presented caprylic, myristic pentadecanoic, and heptadecanoic acids. The last two acids and arachidic acid were dominant in all the samples. The highest content in heptadecanoic was recorded in rapeseed oilcake (RSOC, 22.17%), followed by sesame oilcake (SOC, 15.03%). The highest contents in pentadecanoic and arachidic acids were found in FSOC (4.98%) and SFOC (38.12%), respectively. Among odd-chain FAs, undecanoic and tricosanoic were found only in HSOC, whereas tridecanoic was only found in FSOC. Even-chain FAs such as capric, lauric, eicosadienoic, and lignoceric were absent in SFOC and SOC.

In the tested flour oilcakes, the MUFA content varied between 11.84% (walnut oilcake, WOC) and 54.65% (RSOC). The flours showed a high content of oleic, elaidic, and cis-10 heptadecanoic acids. FSOC contained the highest content of oleic and elaidic acids (34.29%), followed by SOC (29.14%). RSOC and SOC had the highest content of myristoleic acid compared to the other oilcakes, in which the content was below 1%. cis-10 heptadecanoic and gondoic acids were confirmed only in FSOC (a significantly higher amount) and absent in SFOC, RSOC, and SOC. Palmitoleic acid levels in all tested oilcakes were below 1%.

SFOC showed the lowest content of PUFA (24.11%), while the other samples recorded content ranging from 33.19% (FSOC) to 73.41% (WOC). In terms of individual PUFA, only three were detected in all samples: LA, ALA, and cis-11,14,17-eicosatrienoic acid. WOC contained the highest and lowest amounts for LA and PUFA n-3, respectively, from all the oilcakes analyzed. Among the PUFA n-3 acids, ALA was discovered in high concentrations in RSOC (12.48%) and HSOC (6.83%), while the other samples showed content below 2%. In SFOC and RSOC, the presence of γ-linolenic, arachidonic, and cis-5,8,11,14,17-eicosapentenoic acids was not detected. Some detected PUFA were absent in specific oilcakes: cis-11,14-eicosadienoic and cis-8,11,14-eicosatrienoic in HSOC, docosa-hexanoic and nervonic acids in RSOC, and docosa-dienoic acid in WOC. The values are presented in Table 2.

### 2.3. Cluster Analysis

**Figure 1:**
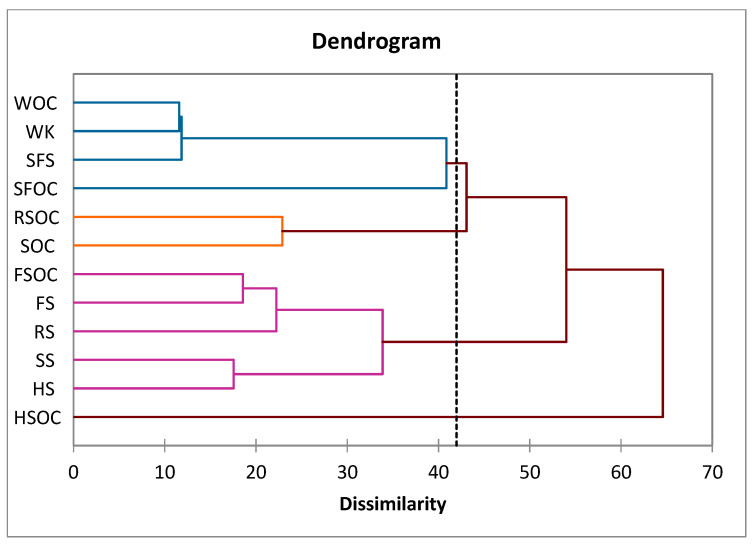
Dendrogram for cluster analyses of oilseeds and oilcakes. SFS, SFOC—sunflower seeds and oilcake, HS, HSOC—hemp seeds and oilcake, RS, RSOC—rape seeds and oilcake, FS, FSOC—flax seeds and oilcake, SS, SOC—sesame seeds an oilcake, WK, WOC—walnut kernels and oilcake.

The samples were grouped as a function of the FA profile. A hierarchical cluster analysis of the FA profile of all the tested oilcakes and oilseeds distinguished four clusters. The first group includes five samples: HS, LS, SS, RS, and LSOC. The second cluster includes four samples (SFS, WK, SFOC, and WOC), the third only one oilcake (HSOC), and the fourth only two (SOC and RSOC) (Figure 1).

According to the results obtained from ANOVA (Table 3), the variables of tridecanoic, pentadecanoic, cis-10-pentadecanoic, cis-10 heptadecanoic, stearic, oileic, elaidic, LA, erucic, cis-11,14-eicosadienoic, docosa-dienoic, docosa-hexanoic, and nervonic acids are not significant (*p* < 0.05) in relation to the cluster membership.

The samples from the first group are characterized by the highest content in γ-linolenic, caprylic, lauric, eicosadienoic, and lignoceric acids, and the lowest content of tricosanoic, arachidonic, and cis-11,14,17-eicosatrienoic acids.

The second group is characterized by the highest content of cis-11,14,17-eicosatrienoic acid. All the other FAs presented the lowest concentrations. The last two groups contain samples with the highest content of ALA and heptadecanoic acids, respectively. 

### 2.4. FA Profile via Fourier Transform Infrared-Attenuated Total Reflection (FTIR-ATR)

**Figure 2:**
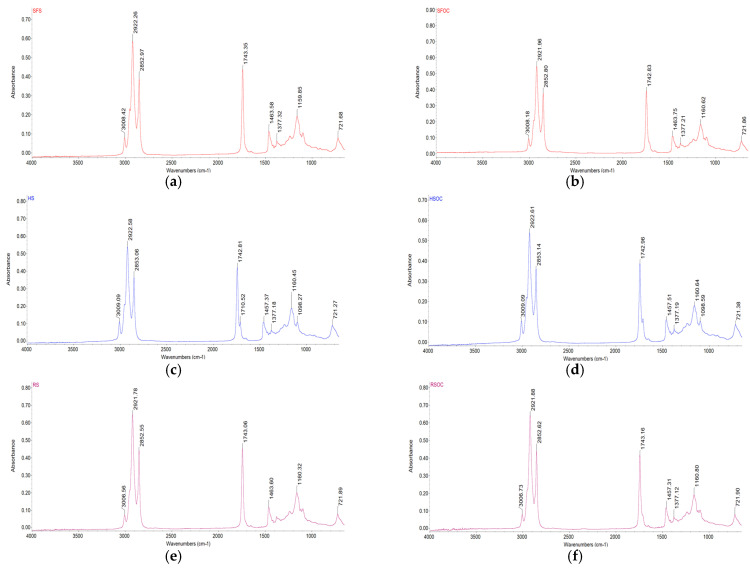

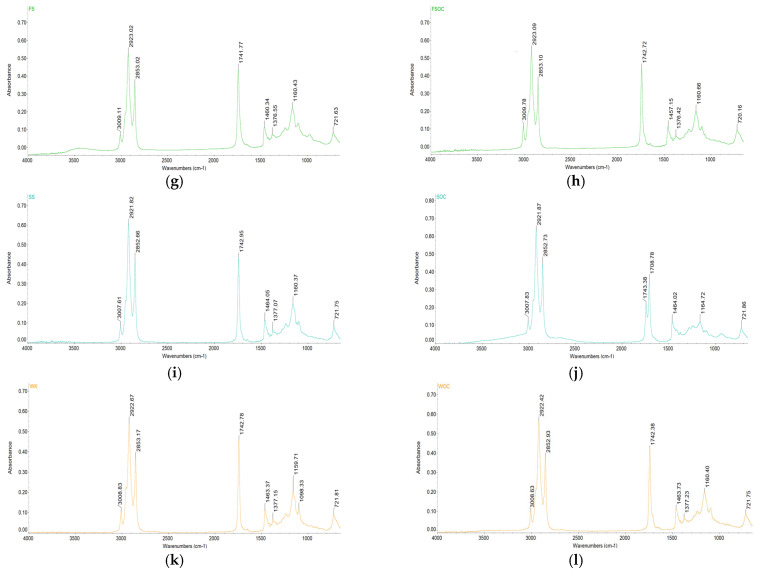
FT-IR spectra of oleaginous seeds and their by-products: (**a**)—SFS, (**b**)—SFOC, (**c**)—HS, (**d**)—HSOC, (**e**)—RS, (**f**)—RSOC, (**g**)—FS, (**h**)—FSOC, (**i**)—SS, (**j**)—SOC, (**k**)—WK, (**l**)—WOC.

FTIR is a method used for the identification of molecular structures, providing detailed information about the molecular bonds observed [34]. To determine the key quality (lipid degradation under oxidative conditions) of vegetable oils, this method has proven to be promising, rapid, and valuable (Figure 2). 

The oils spectra are similar in terms of band position, but their intensity differ from each other depending on the functional groups found in the oil samples [35]. In addition, in WK, WOC, RSOC, HS, and HSOC additional peaks were found at ≈1708 cm^−1^ and 1098 cm^−1^, while in RS and SOC the absence of one peak ≈1376 cm^−1^ was observed.

The region between 3700 and 3100 cm^−1^ contains the stretching vibrations of hydroxyl from water, hydroperoxides, and their degradation products (aldehydes, alcohols, and ketones) [36]. In the region no bands are present for all the oil samples, denoting the absence of oxidation degradation that leads to loss of quality (flavor, aroma, and nutritional value).

The small band at 3006.56–3009.78 cm^−1^ is characteristics of a cis-double bond UFA [37]. The position of this peak indicates the extent of unsaturation (concentration of UFA), which is more evident in FSOC. The band at 1741.77–1743.38 cm^−1^ can be assigned to the stretching vibration of the carbonyl group in triglyceride esters [38]. Spectral changes occurring in the region 3050–2800 and 1745 cm^−1^ serve to monitor the oxidation and adulteration process [39,40]. Two peak ratios can be indicators for lipid peroxidation: 3007/2854 cm^−1^ and 3007/1745. A decreasing trend in these ratios during storage indicates oxidative degradation of the UFA [41]. Sesame and rape seeds and oilcakes exhibited lower values for these ratios, suggesting higher susceptibility to oxidation compared to the other samples.

The absorption bands located at 2921.61–2923.09 cm^−1^ and 2852.55–2853.17 cm^−1^, respectively, are due to the asymmetric and symmetric stretching of methylene groups in the fatty acids [42,43].

Other bands were observed at 1457.15–1464.05 cm^−1^ and 1376.42–1377.32 cm^−1^, attributed to the scissoring vibration of methylene and methyl groups of lipids, proteins, or cholesterol esters, respectively [44].

In the fingerprint region, the bands at 1159.71–1164.72 cm^−1^ and 1098.06–1098.59 cm^−1^ can be attributed to an asymmetric stretching vibration of the C-O ester group; the first peak also confirms the esterification of FA [45,46,47].

All samples presented a band at 720.16–721.89 cm^−1^, produced by a rocking vibration of long chain methylene, characteristic of fatty acids with long carbon skeletons [48,49].

The transformation of cis to trans double bound UFA can be observed by highlighting the changes that occur in regions specific to unsaturation (≈3006 cm^−1^, 967 cm^−1^ and 722 cm^−1^). The appearance of the second band and the decrease of the first and last band indicates the disappearance of the cis double bond and the formation of trans isomers [40]. For all samples, the absence of bands at ≈967 cm^−1^ was observed, denoting the absence of trans UFA.

Several studies have successfully used FTIR to investigate the content of trans FAs in various food matrices. The correlation of the result obtained with those from gas chromatography is accurate (0.98–0.99), demonstrating that it can even detect low levels of trans FA [50,51].

## 3. Discussion and Future Perspectives

The consumption of seeds can exhibit a beneficial effect on human health that can be attributed partly to the lipid components.

Sunflower, sesame, hemp, and walnut seeds and oilcakes have numerous applications in the bakery industry [52,53,54]. The oils extracted from the seeds are also widely used (in margarine, cooking oils formulation, appetizer, and salads) due to their organoleptic properties and other bioactive compounds that provide beneficial physiological effects and oxidative stability [55]. The use of rapeseed as a supplement in the human diet was limited by its high content of toxic erucic acid, so a new cultivar called canola was introduced through breeding [56].

The by-products resulting from oil extraction represent attractive and economical resources for the creation of products with high nutritional value, called functional, satisfying the growing consumer demand for natural healthy foods with improved nutritional characteristics [55,57].

The high polyunsaturated fatty acid content of cakes increases their susceptibility to oxidative degradation [58]. To improve the stability and shelf life before use in food products, natural antioxidants such as tocopherols, polyphenol-rich extracts, or rosemary essential oil can be added [59,60]. This is particularly important when incorporating into protein-rich products, snack bars, meat analogues, plant-based burgers, or fortified flours, where oxidation can negatively affect the sensory and nutritional quality.

The fatty acid profile influences lipid characteristics such as fluidity, melting point, and interfacial behavior [61], which can affect emulsion formation. However, the emulsifying capacity of oilcakes is not determined solely by fatty acids; it results from the synergistic effect of proteins, phospholipids, and other surfactant compounds. Studies show that high levels of polyunsaturated fatty acids (linoleic acid) can improve emulsion stability and antioxidant activity [62]. Thus, oilcakes possess a good emulsifying capacity, which is required in infant formulae, ice cream, sauces, dressings, spreads, and mayonnaise to enhance the texture and mouthfeel [63]. The oleic and linoleic acids from oilcakes contribute to the juiciness, flavor release, and creaminess in meat analogues and dairy alternatives [64,65].

Oil cake recovery offers significant environmental benefits (lower emissions and reduced waste) while also bringing economic advantages (cost savings, new sources of income, and innovation in the food and bio industries). Quantifying these benefits supports stronger policies and investments in recovery strategies [11,66,67].

The level of UFA in all tested samples predominates, which is characteristic of plant oil [68]. Among UFAs, the most representative fatty acids in oilcakes are MUFA n-7, MUFA n-9, and PUFA n-6. The result obtained for the FA profile was in accordance with the results from other studies [55,69,70,71,72,73,74].

The MUFA n-7 fatty acids (palmitoleic acid, cis-vaccenic acid) have a therapeutic effect on diabetes by improving the body sensitivity to insulin. Furthermore, they can help preventing metabolic syndrome and atherosclerosis [75,76]. The cis-MUFA n-9 fatty acids (oleic acid) have a positive effect (comparable to those of ALA and LA) on the serum lipoprotein profile [77].

A PUFA n-6 fatty acid with significant implications for human nutrition and health is γ-linolenic acid. The body transforms it into a bioactive compound (dihomo-gamma-linolenic acid) important in the management of cardiovascular disease, inflammation, and metabolic disorders [78].

The high level of arachidic acid in SFOC can be attributed to a concentration effect caused by the extraction of UFA during pressing. In addition, heat treatments can degrade unsaturated lipids, and mechanical extraction at low temperatures favors the retention of SFA such as arachidic acid [79].

Erucic acid has a toxic effect on human health, the principal target being the heart functions. The major intake source is from rapeseed and *Brassicaceae* [80]. In RSOC, the level decreases from ≈5% to ≈3% after cold pressing. The maximum level set by the European Commission was 5%, making the seeds RSOC, HSOC, SOC, and WOC toxicologically safe for human consumption. Among oilcakes, only FSOC and SFOC need a reduction of this FA before utilization. In infant formulas, the levels need to be reduced below 1% because they are considered a vulnerable category [81]. The use of the investigated samples are disadvantageous and are crucial for updating the dietary recommendations and tolerable daily intakes.

The favorable values obtained for the nutritional evaluation of fat (low IA, TI, and high h/H) compared to other animal origin foods testify to the potential beneficial effect of fat extracted from the tested samples on the cardiovascular system [14,82]. The dietary benchmarks from literature for nutritional indexes that indicate a favorable lipid profile that supports cardiovascular health are higher than 2.5 for the hypocholesterolemic/hypercholesterolemic ratio and below 0.5 for atherogenicity and thrombogenicity indexes [14,83].

The most advantageous parameters for oilseeds can be found in SFS, HS, and WK, while RS was characterized by the highest index values (0.34 and 0.20, respectively) and lowest h/H ratio (4.27).

**Figure 3:**
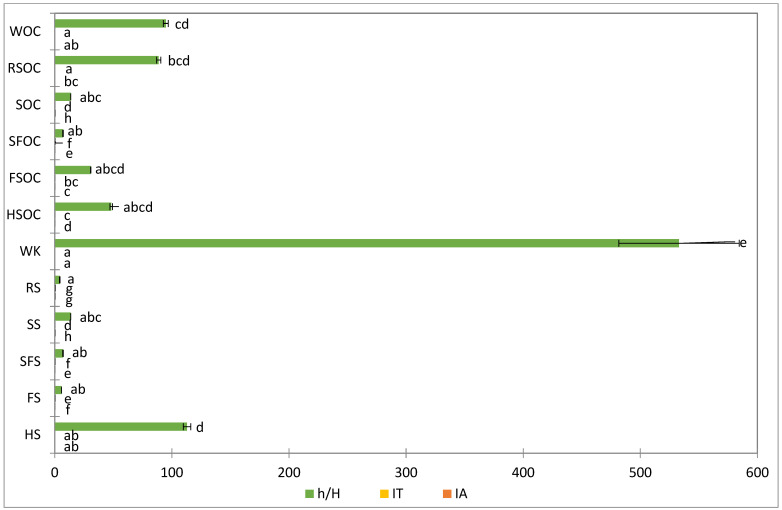
Nutritional indexes of oil seeds and cakes. Different letters means significant (*p* < 0.05) differences among samples via ANOVA, Tukey test post-hoc. SFS, SFOC—sunflower seeds and oilcake, HS, HSOC—hemp seeds and oilcake, RS, RSOC—rape seeds and oilcake, FS, FSOC—flax seeds and oilcake, SS, SOC—sesame seeds an oilcake, WK, WOC—walnut kernels and oilcake.

The most advantageous parameters for oilcakes can be found in WK and RSOC. In contrast, SFOC was characterized by the highest index values (0.13 and 0.29, respectively) and lowest h/H ratio (7.09) (Figure 3).

In maintaining good health, it is pivotal to pay attention to the amount and quality of fat supplied. Modern diets are characterized by a high intake of SFA and trans FA, as well as an imbalance of the ratio of PUFA n-6 and n-3, with excessive intake of n-6 and deficiency of n-3 (10:1 and 20:1) [84]. These can promote a propensity for inflammation and several diseases (diabetes, depression, neurological, and immune disorders) [85]. By including seeds in diets as sources of PUFA n-3, the ratio can be improved [86,87]. Access to healthy dietary fats is unequal, largely due to socioeconomic factors. Because they are more expensive, they are more difficult to obtain for low-income populations. This leads to poorer diet quality, highlighting the need for policies that improve the accessibility of healthy fats [16]. In the present work, the content of PUFA n-3, in particular ALA, is significantly higher (*p* < 0.05) in oilcakes than in seeds. In contrast, among PUFA n-6, linoleic acid has the highest concentration. Of all samples, RSOC and HSOC have the higher content of this FA. The optimal nutritional ratio of LA to ALA ranged from 1:1 to 5:1 [88], and the ratio 3:1 was obtained for our samples. The n-6/n-3 ratio of oilcake reflects a balanced fatty acid profile, aligned with international health guidelines (5:1 to 10:1, the Food and Agriculture Organization and National Institute of Health) and the ideal dietary ratio (≤4:1). This balance is linked to reduced inflammation and better cardiac and metabolic health, highlighting the potential of oilcake as a functional food ingredient.

## 4. Materials and Methods

### 4.1. Samples

The research material comprised twelve groups of plant seeds and nuts with the respective oilcake remaining after cold oil production. The tested samples are presented in Table 4. The chosen seeds were selected because they can be used as functional food ingredients, being sources for proteins, PUFA, and fibers.

Sunflower, hemp, and walnut oilcakes were donated from local factories (PISOK A.B. INTERNATIONAL SRL, NEL-CRIS S.R.L., Suceava, Romania). Flax, sesame and rapeseed oilcakes were purchased from another factory (OLEOMET S.R.L., Bucharest, Romania).

### 4.2. Analytical Methods

Moisture standardization prior to further analysis was achieved with an oven drying method (105 °C, until the mass of samples remained constant) [89].

Lipid content was determined gravimetrically after extraction with petroleum ether (analytical grade, Sigma-Aldrich, St. Louis, MO, USA) for 120 min and solvent evaporation (for 60 min) in an automatic Soxhlet extraction system (Model SER 148, Velp Scientific, Usmate Velate, Italy) [90]. For each sample, three replicates were conducted.

The fatty acid composition was determined using a method described by Petraru et al. [33], which involves the preparation of fatty acids methyl esters (FAMES) via transesterification (firstly, 0.03 g oil was mixed with 2 mL of isooctane, secondly, under vigorous stirring, 0.2 mL methanolic solution of potassium hydroxide 2M was added, and lastly, the supernatant was collected), their separation on a capillary column DB-WAX (30 m × 0.25 mm × 0.25 µm), and their analysis with a gas chromatograph (GC, Agilent Technologies, 6890N GC, Wilmington, NC, USA) with a flame ionization detector (FID).

The initial temperature of the GC oven was 60 °C (for 1 min), increased firstly to 200 °C (10 °C/min and held for 2 min) and then to 220 °C (5 °C/min and held for 20 min). The injector and detector temperature was held at 250 °C. The carrier gas used was helium with a flow rate of 30 mL/min, while air and hydrogen were supplied at flow rates of 450 mL/min and 40 mL/min, respectively. All analyses were done in duplicate [26].

### 4.3. Nutritional Indices

The functional quality of the investigated samples was determined through various indices such as [91]:

- total saturated fatty acids (ΣSFAs);
- total monounsaturated fatty acids (ΣMUFA);
- total polyunsaturated fatty acids (ΣPUFA);
- ratio of total n-6 and n-3 families of polyunsaturated fatty acids (Σn6 PUFA/Σn3 PUFA);
- ratio of polyunsaturated to saturated fatty acids (PUFA/SFA);
- atherogenicity index (IA) indicating the relationship between the sum of SFA and UFA (Equation (1)) considered with pro- and anti-atherogenic potential (capacity to favor/inhibit the accumulation of plaque on the circulatory system), respectively [14];

(1) $$\mathrm{IA}=\frac{C12:0+\left(4\;\times \;C14:0\right)+C16:0}{\Sigma \mathrm{MUFA}+\Sigma \mathrm{PUFA}}$$

- thrombogenicity index (IT, Equation (2)) indicating the relationship between the sum of pro- and anti-thrombogenic potential (capacity to favor/inhibit the formation of clots in blood vessels) [92];

(2) $$\mathrm{IT}\;=\frac{C14:0+C16:0+C18:0}{0.5\;\times \;\left(\sum \mathrm{MUFA}+\;\sum n6\;\mathrm{PUFA}\right)+\left(3\;\times \;\sum n3\;\mathrm{PUFA}\right)+\left(\sum n6\;\mathrm{PUFA}/\sum n3\;\mathrm{PUFA}\right)}$$

- hypocholesterolemic to hyercholesterolemic ratio (h/H) indicating the relationship between hypocholesterolemic and hypercholesterolemic FA, calculated according to Equation (3) [93].

(3) $$h/H=\frac{C18:1+C18:2+C18:3(n-3)}{C12:0+C14:0+C16:0}$$

### 4.4. FTIR Analysis of Oilseeds and Oilcakes FA Profiles

The FTIR spectra was recorded with a spectrometer (Nicolet iS20, Thermo Scientific, Karlsruhe, Germany) combined with an attenuated total reflectance accessory and a diamond crystal. The data acquisition (32 scans) was carried at a resolution of 4 cm^−1^ in the region between 400 and 4000 cm^−1^. The background spectrum of air was used to normalize the spectra baseline [45].

### 4.5. Statistical Analysis

All analyses regarding the FA profile were performed in duplicate and the measured values are expressed as mean ± standard deviation. One-way ANOVA, followed by Tukey’s test, was performed using XLSTAT software (Addinsoft, New York, NY, USA, trial version) to assess the significant difference at *p*-value < 0.05.

In order to group the investigated seeds and oilcake resulting after cold oil extraction, according to their FA profile, a hierarchical cluster analysis was performed. The grouping was performed with the Ward agglomeration procedure using the Euclidean distance as a function of the distance. The cut-off point was established at 42%. The differences among clusters were evaluated by applying a one-way ANOVA analysis with Tukey’s test post-hoc using XLSTAT software (trial version).

## 5. Conclusions

Lipids are indispensable components in human nutrition providing energy and numerous health benefits, including prevention and/or treatment of chronic diseases.

The fatty acid composition of both the seeds and the resulting oilcakes was determined. In SFOC the predominant fatty acid was arachidonic, in SR, SC, and SS linoleic and linolelaidic, and in SI oleic and elaidic acids. The highest content of saturated fatty acids was in sunflower oilcake (55.85%). The n-3/n-6 PUFA ratios in the oilcakes (values ranging from 3.17 to 17.92) were much lower than those obtained for seeds, indicating that they have a beneficial nutritional profile in combating cardiovascular risk factors.

To discriminate between samples from the same category or by-products obtained from the same raw material, a cluster analysis was performed. Only hemp seeds have a unique fatty acid profile and constitute a category in themselves.

Despite their affordable price and potential benefits, oilcakes are not used inappropriately in industrial applications, especially in the food sector. There are studies in the literature on replacing traditional ingredients with various oilcakes for the production of new food products, but these are only conceptualizations. The progress made has some limitations in terms of process scalability and regulatory acceptance.

## Figures and Tables

**Table 1:** Fat content and fatty acid methyl esters profile of oleaginous seeds.

	SFS	HS	RS *	FS *	SS	WK
**Lipids, %**	64.76 ± 0.28 ^a^	25.54 ± 0.53 ^e^	35.22 ± 1.39 ^d^	27.74 ± 0.48 ^e^	46.23 ± 0.43 ^c^	59.55 ± 2.23 ^b^
**SFA, %**						
Caprylic acid (C8:0)	0.33 ± 0.01 ^d^	1.12 ± 0.03 ^b^	0.41 ± 0.01 ^c^	0.32 ± 0.01 ^d^	0.25 ± 0.01 ^e^	0.13 ± 0.01 ^f^
Capric acid (C10:0)	nd	0.27 ± 0.01 ^e^	0.36 ± 0.03 ^d^	nd	nd	nd
Lauric acid (C12:0)	nd	0.26 ± 0.00 ^e^	0.37 ± 0.02 ^d^	0.35 ± 0.01 ^d^	nd	nd
Myristic acid (C14:0)	0.43 ± 0.00 ^d^	0.41 ± 0.02 ^d^	0.55 ± 0.04 ^c^	0.46 ± 0.02 ^d^	0.28 ± 0.01 ^e^	0.18 ± 0.02 ^f^
Pentadecanoic acid (C15:0)	1.19 ± 0.01 ^e^	4.72 ± 0.12 ^d^	4.45 ± 0.28 ^d^	11.86 ± 0.04 ^c^	0.53 ± 0.03 ^f^	0.27 ± 0.02 ^f^
Palmitic acid (C16:0)	nd	nd	12.27 ± 0.87 ^c^	6.79 ± 0.03 ^e^	8.44 ± 0.06 ^d^	nd
Heptadecanoic acid (C17:0)	1.30 ± 0.03 ^d^	1.72 ± 0.06 ^c^	nd	7.80 ± 0.14 ^b^	1.04 ± 0.01 ^d^	0.62 ± 0.04 ^e^
Stearic acid (C18:0)	1.20 ± 0.08 ^d^	0.76 ± 0.00 ^e^	1.08 ± 0.03 ^d^	1.65 ± 0.08 ^c^	0.39 ± 0.00 ^f^	0.36 ± 0.00 ^f^
Arachidic acid (C20:0)	20.60 ± 0.21 ^b^	1.04 ± 0.05 ^e^	nd	8.50 ± 0.06 ^c^	3.15 ± 0.21 ^d^	nd
Heneicosanoic acid (C21:0)	nd	nd	0.38 ± 0.01 ^c^	0.52 ± 0.00 ^b^	0.26 ± 0.01 ^d^	0.18 ± 0.01 ^e^
Eicosadienoic acid (C22:0)	nd	0.14 ± 0.00 ^e^	0.21 ± 0.01 ^cd^	0.15 ± 0.00 ^d^	0.23 ± 0.01 ^c^	nd
Tricosanoic acid (C23:0)	nd	2.92 ± 0.11 ^e^	4.15 ± 0.03 ^d^	4.05 ± 0.03 ^d^	nd	nd
Lignoceric acid (C24:0)	0.20 ± 0.01 ^c^	0.14 ± 0.00 ^e^	0.17 ± 0.01 ^cd^	0.14 ± 0.00 ^de^	nd	nd
**MUFA, %**						
Myristoleic acid (C14:1, n-5)	0.15 ± 0.00 ^de^	0.18 ± 0.01 ^cd^	0.21 ± 0.01 ^c^	0.19 ± 0.01 ^c^	0.14 ± 0.01 ^e^	0.06 ± 0.01 ^f^
cis-10-pentadecanoic acid (C15:1, n-5)	1.10 ± 0.01 ^d^	nd	6.21 ± 0.03 ^c^	nd	1.01 ± 0.01 ^e^	nd
Palmitoleic acid (C16:1, n-7)	0.40 ± 0.01 ^c^	0.51 ± 0.02 ^b^	0.52 ± 0.01 ^b^	0.19 ± 0.01 ^e^	0.34 ± 0.01 ^d^	0.12 ± 0.01 ^f^
cis-10 heptadecanoic acid (C17:1)	nd	1.55 ± 0.01 ^e^	nd	4.34 ± 0.10 ^c^	nd	1.72 ± 0.01 ^d^
Oleic acid + Elaidic acid (C18:1, cis + trans, n-9)	12.00 ± 0.25 ^e^	28.22 ± 0.30 ^b^	10.24 ± 0.49 ^f^	20.64 ± 0.07 ^d^	33.76 ± 0.34 ^a^	26.47 ± 0.31 ^c^
Gondoic acid (C20:1, n-9)	nd	0.78 ± 0.04 ^e^	nd	1.90 ± 0.01 ^c^	1.51 ± 0.01 ^d^	nd
Erucic acid (C22:1, n-9)	4.88 ± 0.03 ^e^	4.21 ± 0.04 ^d^	5.57 ± 0.18 ^b^	4.89 ± 0.01 ^c^	3.88 ± 0.04 ^e^	1.94 ± 0.03 ^f^
**PUFA, %**						
Linoleic acid + linolelaidic acid (C18:2, cis + trans, n-6)	54.50 ± 0.28 ^c^	46.11 ± 0.23 ^d^	44.72 ± 0.64 ^d^	18.03 ± 0.19 ^f^	39.93 ± 0.04 ^e^	65.68 ± 0.67 ^b^
γ-Linolenic acid (C18:3, n-6)	nd	nd	0.64 ± 0.00 ^d^	1.05 ± 0.06 ^c^	nd	0.33 ± 0.02 ^e^
α-Linolenic acid (C18:3, n-3)	1.25 ± 0.01 ^c^	0.72 ± 0.03 ^e^	1.17 ± 0.07 ^cd^	2.46 ± 0.02 ^b^	1.03 ± 0.04 ^d^	0.37 ± 0.02 ^f^
cis-11,14-eicosadienoic acid (C20:2, n-6) + cis-8,11,14-eicosatrienoic acid (C20:3, n-6)	1.00 ± 0.00 ^c^	0.28 ± 0.04 ^ef^	1.26 ± 0.01 ^b^	0.43 ± 0.01 ^d^	0.35 ± 0.02 ^e^	0.25 ± 0.00 ^f^
cis-11,14,17-eicosatrienoic acid (C20:3, n-3)	0.66 ± 0.01 ^c^	0.15 ± 0.01 ^e^	0.31 ± 0.01 ^d^	0.25 ± 0.01 ^e^	0.14 ± 0.00 ^f^	0.33 ± 0.02 ^d^
Arachidonic acid (C20:4, n-6)	0.31 ± 0.00 ^e^	0.16 ± 0.01 ^f^	0.46 ± 0.06 ^d^	0.28 ± 0.01 ^e^	0.14 ± 0.00 ^f^	0.27 ± 0.02 ^e^
cis-5,8,11,14,17-eicosapentenoic acid (C20:5, n-3)	0.25 ± 0.01 ^c^	0.15 ± 0.00 ^d^	nd	0.15 ± 0.00 ^c^	nd	0.06 ± 0.00 ^e^
Docosa-dienoic acid (C22:2, n-6)	0.30 ± 0.01 ^d^	0.72 ± 0.04 ^b^	nd	0.18 ± 0.00 ^e^	0.58 ± 0.00 ^c^	nd
Docosa-hexanoic acid (C22:6, n-3) + nervonic acid (C24:1, n-9)	3.08 ± 0.03 ^d^	2.75 ± 0.07 ^de^	4.31 ± 0.11 ^c^	2.45 ± 0.04 ^e^	2.60 ± 0.01 ^e^	0.65 ± 0.05 ^f^

SFS—sunflower seeds, HS—hemp seeds, RS—rape seeds, FS—flax seeds, SS—sesame seeds, WK—walnut kernels, nd—not detected. * Values presented in other author studies: [32,33]. Different superscript letters after values means significant difference (ANOVA, Tukey test, *p* < 0.05%).

**Table 2:** Fat content and fatty acid methyl esters profile of different oilcakes.

	SFOC	HSOC	RSOC *	FSOC *	SOC	WOC
**Lipids, %**	15.48 ± 0.22 ^b^	9.96 ± 0.02 ^e^	13.24 ± 0.03 ^c^	11.61 ± 0.40 ^d^	24.66 ± 0.10 ^a^	9.63 ± 0.08 ^e^
**SFA, %**						
Caprylic acid (C8:0)	0.59 ± 0.04 ^d^	1.33 ± 0.04 ^b^	0.65 ± 0.04 ^d^	1.01 ± 0.04 ^c^	0.29 ± 0.00 ^e^	0.16 ± 0.01 ^f^
Capric acid (C10:0)	nd	0.56 ± 0.02 ^b^	0.23 ± 0.01 ^d^	0.37 ± 0.02 ^c^	nd	0.12 ± 0.01 ^e^
Undecanoic acid (C11:0)	nd	0.31 ± 0.00 ^f^	nd	nd	nd	nd
Lauric acid (C12:0)	nd	nd	0.21 ± 0.00 ^d^	0.40 ± 0.01 ^c^	nd	0.13 ± 0.01 ^e^
Tridecanoic acid (C13:0)	nd	nd	nd	0.23 ± 0.00 ^f^	nd	nd
Myristic acid (C14:0)	0.68 ± 0.01 ^d^	0.98 ± 0.03 ^c^	0.48 ± 0.03 ^e^	0.61 ± 0.01 ^d^	4.26 ± 0.03 ^b^	0.33 ± 0.02 ^f^
Pentadecanoic acid (C15:0)	7.50 ± 0.14 ^c^	7.29 ± 0.41 ^c^	5.45 ± 0.35 ^d^	4.98 ± 0.14 ^d^	1.87 ± 0.05 ^e^	0.60 ± 0.01 f
Palmitic acid (C16:0)	2.95 ± 0.08 ^b^	nd	nd	0.92 ± 0.03 ^c^	0.57 ± 0.01 ^d^	0.31 ± 0.00 ^e^
Heptadecanoic acid (C17:0)	1.90 ± 0.08 ^f^	14.03 ± 0.04 ^d^	22.17 ± 0.26 ^c^	1.31 ± 0.07 ^f^	15.03 ± 0.07 ^d^	10.13 ± 0.43 ^e^
Stearic acid (C18:0)	4.11 ± 0.15 ^b^	2.39 ± 0.13 ^c^	0.47 ± 0.02 ^e^	1.16 ± 0.01 ^d^	nd	0.36 ± 0.00 ^e^
Arachidic acid (C20:0)	38.12 ± 0.17 ^d^	nd	nd	nd	nd	2.04 ± 0.14 ^e^
Heneicosanoic acid (C21:0)	nd	0.93 ± 0.04 ^b^	0.50 ± 0.04 ^d^	0.69 ± 0.01 ^c^	0.33 ± 0.01 ^e^	0.36 ± 0.01 ^e^
Eicosadienoic acid (C22:0)	nd	nd	0.10 ± 0.00 ^e^	0.23 ± 0.01 ^c^	nd	0.12 ± 0.00 ^d^
Tricosanoic acid (C23:0)	nd	6.47 ± 0.04 ^e^	nd	nd	nd	nd
Lignoceric acid (C24:0)	nd	0.34 ± 0.00 ^f^	nd	0.21 ± 0.00 ^f^	nd	nd
**MUFA, %**						
Myristoleic acid(C14:1, n-5)	0.38 ± 0.01 ^e^	0.64 ± 0.00 ^d^	1.71 ± 0.06 ^c^	0.31 ± 0.01 ^ef^	2.17 ± 0.10 ^b^	0.18 ± 0.01 ^f^
cis-10-pentadecanoic acid (C15:1, n-5)	1.17 ± 0.04 ^d^	nd	nd	4.27 ± 0.04 ^c^	0.36 ± 0.01 ^e^	nd
Palmitoleic acid (C16:1, n-7)	0.32 ± 0.00 ^f^	0.74 ± 0.00 ^d^	0.65 ± 0.04 ^e^	0.68 ± 0.01 ^de^	0.70 ± 0.00 ^d^	0.39 ± 0.01 ^f^
cis-10 heptadecanoic acid (C17:1)	nd	nd	nd	8.06 ± 0.16 ^d^	nd	4.28 ± 0.09 ^e^
Oleic acid + elaidic acid (C18:1, cis + trans, n-9)	9.41 ± 0.57 ^e^	17.66 ± 0.13 ^d^	9.12 ± 0.16 ^e^	34.29 ± 0.05 ^b^	29.14 ± 0.37 ^c^	2.54 ± 0.00 ^f^
Gondoic acid (C20:1, n-9)	nd	3.59 ± 0.08 ^d^	nd	1.08 ± 0.03 ^e^	nd	1.16 ± 0.05 ^e^
Erucic acid (C22:1, n-9)	8.75 ± 0.35 ^b^	5.16 ± 0.23 ^d^	3.75 ± 0.14 ^ef^	5.98 ± 0.04 ^c^	4.27 ± 0.04 ^e^	3.29 ± 0.11 ^f^
**PUFA, %**						
Linoleic acid + Linolelaidic acid (C18:2, cis + trans, n-6)	15.03 ± 0.04 ^f^	22.74 ± 0.06 ^e^	39.63 ± 0.67 ^c^	22.91 ± 0.16 ^e^	34.47 ± 0.44 ^d^	70.22 ± 1.22 ^b^
γ-Linolenic acid (C18:3, n-6)	nd	1.49 ± 0.01 ^c^	nd	1.43 ± 0.01 ^d^	0.48 ± 0.00 ^e^	nd
α-Linolenic acid (C18:3, n-3)	1.16 ± 0.06 ^e^	6.83 ± 0.04 ^b^	12.48 ± 0.03 ^a^	1.65 ± 0.02 ^d^	1.92 ± 0.06 ^c^	0.21 ± 0.01 ^f^
cis-11,14-eicosadienoic acid (C20:2, n-6) + cis-8,11,14-eicosatrienoic acid (C20:3, n-6)	0.58 ± 0.01 ^e^	nd	1.75 ± 0.07 ^c^	0.87 ± 0.01 ^d^	0.20 ± 0.01 ^f^	1.07 ± 0.08 ^d^
cis-11,14,17-eicosatrienoic acid (C20:3, n-3)	0.56 ± 0.00 ^c^	0.35 ± 0.01 ^de^	0.40 ± 0.03 ^d^	0.34 ± 0.0 ^e^	0.26 ± 0.00 ^f^	0.66 ± 0.02 ^b^
Arachidonic acid (C20:4, n-6)	nd	0.41 ± 0.04 ^e^	nd	0.33 ± 0.01 ^e^	nd	0.56 ± 0.04 ^d^
cis-5,8,11,14,17-eicosapentenoic acid (C20:5, n-3)	nd	0.43 ± 0.01 ^c^	nd	0.35 ± 0.01 ^d^	nd	0.11 ± 0.01 ^e^
Docosa-dienoic acid (C22:2, n-6)	0.32 ± 0.01 ^d^	0.31 ± 0.01 ^d^	0.25 ± 0.014 ^e^	0.30 ± 0.30 ^d^	0.48 ± 0.01 ^c^	nd
docosa-hexanoic acid (C22:6, n-3) + nervonic acid (C24:1, n-9)	6.47 ± 0.38 ^c^	5.02 ± 0.03 ^d^	nd	5.03 ± 0.03 ^d^	3.21 ± 0.04 ^e^	0.59 ± 0.04 ^f^

SFOC—sunflower oilcake, HSOC—hempseed oilcake, RSOC—rapeseed oilcake, FSOC—flaxseed oilcake, SOC—sesame oilcake, WOC—walnut oilcake, nd—not detected. * Values presented in other author studies: [32,33]. Different superscript letters after values means significant difference (ANOVA, Tukey test, *p* < 0.05%).

**Table 3:** Oil seed and cake groups by fatty acid methyl esters profile.

Characteristic	Mean				*p*-Value
	1	2	3	4	
C8:0	0.622 ^a^	0.299 ^a^	1.330 ^b^	0.468 ^a^	0.002 *
C10:0	0.198 ^a^	0.030 ^a^	0.555 ^b^	0.113 ^a^	0.001 *
C11:0	0.000 ^a^	0.000 ^a^	0.310 ^a^	0.000 ^a^	0.000 *
C12:0	0.273 ^b^	0.032 ^a^	0.000 ^a^	0.105 ^ab^	0.001 *
C13:0	0.246 ^a^	0.000 ^a^	0.000 ^a^	0.000 ^a^	0.228
C14:0	0.457 ^a^	0.403 ^a^	0.980 ^ab^	2.370 ^b^	0.006 *
C14:1, n-5	0.205 ^a^	0.191 ^a^	0.640 ^b^	1.938 ^c^	0.000 *
C15:0	5.306 ^a^	2.386 ^a^	7.290 ^a^	3.655 ^a^	0.179
C15:1, n-5	2.296 ^a^	0.566 ^a^	0.000 ^a^	0.183 ^a^	0.112
C16:0	5.685 ^a^	0.814 ^a^	0.000 ^a^	0.283 ^a^	0.014 *
C16:1, n-7	0.450 ^ab^	0.305 ^a^	0.740 ^b^	0.660 ^b^	0.001 *
C17:0	2.393 ^a^	3.485 ^a^	14.030 ^b^	18.598 ^b^	0.000 *
C17:1	2.791 ^a^	1.499 ^a^	0.000 ^a^	0.000 ^a^	0.209
(C18:0	1.009 ^a^	1.505 ^a^	2.390 ^a^	0.233 ^a^	0.096
C18:1, cis + trans, n-9	25.830 ^b^	12.601 ^a^	17.655 ^ab^	19.128 ^ab^	0.059
C18:2, cis + trans, n-6	34.340 ^a^	50.355 ^a^	22.740 ^a^	37.048 ^a^	0.105
C18:3, n-6	0.625 ^ab^	0.082 ^a^	1.485 ^b^	0.240 ^a^	0.002 *
C18:3, n-3	1.404 ^a^	0.745 ^a^	6.830 ^b^	7.200 ^b^	0.000 *
C20:0	2.557 ^a^	15.189 ^a^	0.000 ^a^	0.000 ^a^	0.040 *
C20:1, n-9	1.058 ^b^	0.289 ^a^	3.585 ^c^	0.000 ^a^	0.000 *
C20:2, n-6 + C20:3, n-6	0.635 ^a^	0.724 ^a^	0.000 ^a^	0.978 ^a^	0.168
C20:3, n-3	0.236 ^a^	0.549 ^b^	0.345 ^ab^	0.330 ^a^	0.000 *
C20:4, n-6	0.274 ^b^	0.284 ^b^	0.410 ^b^	0.000 ^a^	0.014 *
C20:5, n-3	0.139 ^a^	0.091 ^a^	0.425 ^b^	0.000 ^a^	0.001 *
C21:0	0.370 ^a^	0.134 ^a^	0.930 ^b^	0.415 ^a^	0.000 *
C22:0	0.199 ^b^	0.030 ^a^	0.000 ^a^	0.051 ^a^	0.000 *
C22:1, n-9	4.902 ^a^	4.445 ^a^	5.160 ^a^	4.010 ^a^	0.792
C22:2, n-6	0.357 ^a^	0.153 ^a^	0.305 ^a^	0.363 ^a^	0.224
C22:6, n-3 + C24:1, n-9)	3.425 ^a^	2.695 ^a^	5.020 ^a^	1.605 ^a^	0.175
C23:0	2.229 ^b^	0.000 ^a^	6.470 ^c^	0.000 ^a^	0.000 *
C24:0	0.134 ^b^	0.049 ^a^	0.340 ^c^	0.000 ^a^	0.000 *

* Values presented are significant at *p* < 0.05. Different superscript letters after values means significant difference (ANOVA, Tukey test, *p* < 0.05%).

**Table 4:** Research material.

Name	Family	Abbreviation
Sunflower: whole seeds (*Helianthus annus*) oilcake (pellets)	*Asteraceae*	SFS SFOC
Hemp: whole seeds (*Cannabis sativa*) oilcake (pellets)	*Cannabaceae*	HS HSOC
Flax: whole seeds (*Linum uitatissimum*) oilcake (ground flour)	*Linaceae*	FS FSOC
Rape: whole seeds (*Brassica napus*) oilcake (ground flour)	*Brassicaceae*	RS RSOC
Sesame: whole seeds (*Sesamum indicum*) oilcake (ground flour)	*Pedaliaceae*	SS SOC
Walnut: kernels (*Junglas Regia*) oilcake (ground flour)	*Juglandaceae*	WK WOC

## Data Availability

The original contributions presented in this study are included in the article. Further inquiries can be directed to the corresponding author.

## Abbreviations

The following abbreviations are used in this manuscript:

ALA	α-Linolenic acid
FA	Fatty acids
FS	Flax seeds
FSOC	Flaxseed oilcake
HS	Hemp seeds
HSOC	Hempseed oilcake
h/H	hypocholesterolemic to hyercholesterolemic ratio
IA	Atherogenicity index
IT	Thrombogenicity index
LA	Linoleic acid
MUFA	Monounsaturated fatty acids
PUFA	Polyunsaturated fatty acids
RS	Rape seeds
RSOC	Rapeseed oilcake
SFA	Saturated fatty acids
SFOC	Sunflower oilcake
SFS	Sunflower seeds
SOC	Sesame oilcake
SS	Sesame seeds
WOC	Walnut oilcake
WK	Walnut kernels
